# Role of FGF19 induced FGFR4 activation in the regulation of glucose homeostasis

**DOI:** 10.18632/aging.100108

**Published:** 2009-12-09

**Authors:** Xinle Wu, Yang Li

**Affiliations:** Amgen Inc., South San Francisco, CA 94080, USA

**Keywords:** fibroblast growth factors, FGF19, FGF21, FGF23, aging, diabetes, metabolic diseases, insulin

## Abstract

FGF19, FGF21, and FGF23 form a unique subfamily of fibroblast growth factors.  Because they contain intra-molecular disulfide bonds and show reduced affinity toward heparan sulfate located in the extracellular space, it is thought that, in contrast to other FGFs, they function as endocrine hormones. FGF23 and its co-receptor αKlotho are involved in the control of aging, but it is not known if the same holds true for FGF19, which can also signal through αKlotho. However, considerable evidence supports a role for FGF19 in controlling various aspects of metabolism.  We have recently fully characterized FGF19/FGFR/co-factor interactions and signaling, and in the current manuscript discuss the contribution of the FGF19/FGFR4 axis to bile acid and glucose regulation.

The fibroblast growth factors (FGFs) family is composed of 22 members that are grouped into 7 subfamilies [1].  Most FGF family members are considered to be paracrine factors, and have been shown to be involved in the processes of development, transformation, and angiogenesis [2-4].  However, the FGF19 subfamily members, which include FGF19, 21, and 23, have recently been shown to function in an endocrine manner and to regulate physiological processes that include glucose, lipid, and energy metabolism, as well as bile acid and serum phosphate homeostasis [5].  One key difference between the FGF19 subfamily and other FGF proteins is their weak affinity toward heparan sulfate of the pericellular space.  This weak affinity allows FGF19 subfamily members to escape from the extracellular compartment into circulation and to function as endocrine hormones [5,6].  Whereas heparan sulfate is used by other FGFs to form high-affinity interactions with FGF receptors (FGFRs), the FGF19 subfamily members instead use single-transmembrane containing Klotho proteins to facilitate their interactions with FGFRs, which compensates for the reduced affinity of FGF19 subfamily members toward heparan sulfates and FGFRs [6].

Two related Klotho proteins, αKlotho and βKlotho, both contain two homologous extracellular domains that share sequence homology to the β-glucosidase of bacteria and plants [7,8].  FGF21 and FGF23 selectively use βKlotho and αKlotho as co-receptors, respectively, while FGF19 can function through either co-receptor*in vitro* [9].  The FGF23-αKlotho axis regulates systemic phosphate, calcium, and vitamin D homeostasis (Figure 1, [10]).  In addition, FGF23-αKlotho is also involved in the control of aging.  Mice over-expressing αKlotho protein live longer than normal mice and manifest a delay in many effects of old age, including weakening of the bone, clogging of the arteries and loss of muscle fitness [7].  Like FGF23, FGF19 can also activate FGFRs via αKlotho *in vitro *[11], however, the physiological significance of this observation is unclear because FGF19 and FGF23 do not appear to share overlapping phenotypes [11,13].

**Figure 1.**
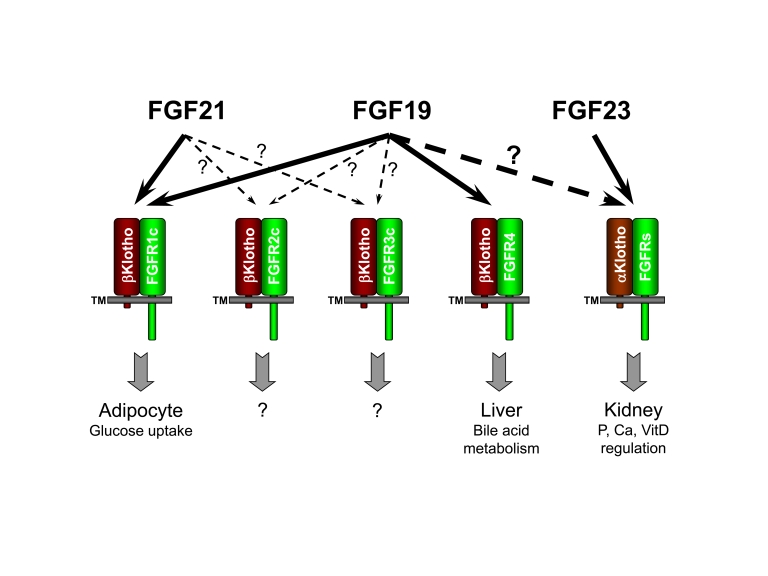
FGF19 subfamily receptor specificity and functions. "?" indicates unresolved research areas associated with the physiological significance of the observed FGF/receptor interactions.

Therefore, determining whether the FGF19-αKlotho axis may also regulate phosphate and vitamin D homeostasis and play a role in controlling the aging process requires further study.

Interactions between βKlotho and FGF19 or FGF21 as well as between βKlotho and FGFRs have been clearly demonstrated by several research groups [6,9,11,14]. Activation of FGFR isoforms 1c, 2c and 3c signaling by FGF19 or FGF21 only occurred when βKlotho is present, which confirms the requirement for βKotho as the co-receptor for these FGFs and is consistent with  the ability of βKlotho to interact with these receptor isoforms [6,14,16].  FGFR isoforms 1b, 2b, and 3b are not activated by FGF19 or FGF21 in the presence of βKlotho presumably due to the inability of βKlotho to interact with these receptor isoforms [6,14,15].  FGFR4 is not activated by FGF21 even though FGFR4 interacts with βKlotho [14,16].  However, FGFR4 can be activated by FGF19 in either the presence or absence of βKlotho [16].  The unique βKlotho independent activity of FGF19 on FGFR4 is partly due to its residual affinity toward heparan sulfate, although its affinity is much weaker compared with canonical FGFs.

One important consequence of the FGF19 subfamily's reliance on a or βKlotho instead of heparan sulfates as co-receptors is that their target tissues are  further limited by the expression pattern of these co-receptor proteins.  Since co-factor heparan sulfates are ubiquitous, canonical FGFs  will activate any tissue as long as their targeting FGFR isoforms are present.  In contrast, FGF19, 21 and 23 typically will only activate tissues where both their targeting FGFR isoforms and a or βKlotho are present.  For example, since βKlotho is primarily expressed in adipose tissue and liver, this expression pattern limits the potential target tissues for FGF21 action.  Since the predominant FGFR residing in the liver is FGFR4, which can not be activated by FGF21, the direct target tissue for FGF21 has been proposed to be further limited to only adipose tissue where FGFR1c and 2c are the major receptors [14,15].  *In vivo* experiments provided support for this hypothesis.  Specifically, when skeletal muscle, adipose tissue, kidney and liver were excised from mice treated with recombinant FGF21, elevated phospho-ERK levels, which represents activation of FGFR signaling, were only observed in lysates of adipose tissue [14,15].

Unlike FGF21, FGF19 is able to activate FGFR4 in addition to FGFRs 1c, 2c, and 3c; therefore, the liver is potentially a direct target tissue of FGF19 in addition to adipocytes.  Consistent with this hypothesis, ERK phophorylation levels increased in both mouse adipose tissue and liver after recombinant FGF19 treatment [14]. In addition,  it has been proposed that FGF19 activates FGFR4 to inhibit liver Cyp7A1 mRNA expression levels and that this inhibition is mediated through SHP and HNF4α [17,18]. Cyp7A1 encodes cholesterol 7 α-hydroxylase, which is the key enzyme in the bile acid biosynthesis pathway [18].  Decreased Cyp7A1 mRNA levels will lead to reduced production of bile acid from cholesterol.  In FGFR4 knockout mice, FGF19 no longer affects Cyp7A1 mRNA levels in the liver, confirming the role of FGFR4 signaling in mediating FGF19 inhibition of Cyp7A1 expression [18].  Genetic ablation of βKlotho gene in mice increases bile acid synthesis and Cyp7A1 expressing level, probably due to the weakened activation of liver FGFR4 [19].  These results are consistent with a role for FGF19 in the regulation bile acid synthesis from liver.

Recently, there has been new evidence suggesting that FGFR4 is also involved in phenotypes related to the metabolic syndrome.  For example, FGFR4-deficient mice that were fed a regular diet displayed hyper-lipidemia, glucose intolerance and insulin resistance as well as increased weight gain compared with wild type litter mates.  Restoration of FGFR4 in the livers of FGFR4 deficient mice decreased plasma lipid levels [20].  FGFR4 has also been implicated in insulin regulation with FoxO1 as a key node in integrating the FGF and insulin signaling pathways [21].  Since recombinant FGF19 improves dyslipidemia and insulin sensitivity and reduces adiposity in diet-induced obese (DIO) mice, it is important to understand how liver and FGFR4 contribute to lipid and glucose homeostasis in addition to bile acid biosynthesis regulation.  However, studying the contribution of the liver FGF19/FGFR4 pathway is complicated by the fact that FGF19 can also induce signaling in other tissues.  The ability of FGF19 to activate both adipose tissue and liver, makes it difficult to assess the contribution of each target tissue individually.

It was known that the C-terminal tail of FGF19 is important for co-receptor interaction [9].  Amino acid sequence alignments demonstrate the absence of the FGF19 C-terminal βKlotho binding domain in canonical FGFs such as FGF1 and FGF2, which do not require co-receptors such as βKlotho.  It is conceivable that the C-terminal tail in FGF19 was acquired during evolution to compensate for its dramatically weakened affinity toward heparan sulfates. However, the fact that FGF19 can activate FGFR4 signaling in the absence of βKlotho suggests that at least for FGFR4, the affinity between FGF19 and heparan sulfates might be sufficient to induce receptor activation under certain conditions.  If that is the case, deletion of the C-terminal βKlotho-binding domain should not affect its ability to activate FGFR4 because the predicted heparan sulfates interacting regions are not located in the C-terminal tail of FGF19 [6].  We therefore constructed and purified a truncated FGF19, FGF19dCTD, without its C-terminal βKlotho-interacting domain and characterized its activity  *in vitro* and *in vivo*.  An *in vitro* receptor specificity assay confirmed that FGF19dCTD is still able to activate FGFR4 but not other FGFRs even in the presence of βKlotho.  FGF19dCTD thus became an FGFR4 specific activator.  In mice injected with FGF19dCTD, ERK phosphorylation was observed only in liver (where FGFR4 expression is predominant) but not in the fat tissues (where FGFR1c and 2c expressions are predominant).  Consistent with increased liver ERK phosphorylation after FGF19dCTD treatment, liver CYP7A1 mRNA expression levels were also suppressed in mice injected with FGF19dCTD.  However, the ability to reduce plasma glucose levels and to improve glucose tolerance has been lost with FGF19dCTD, suggesting a limited contribution from direct activation of FGFR4 toward glucose regulation.  Since other than liver, βKlotho is predominantly expressed in adipose tissue and pancreas, it is reasonable to speculate that direct activation of these tissues by FGF19 is the major mechanism contributing to the regulation of glucose homeostasis.

There are still unanswered questions regarding the mechanisms by which FGF19 and FGF21 regulate glucose metabolism and improve insulin sensitivity; however, data from a recently published study suggest that adipocytes may play an important role in these processes.  Both FGF19 and FGF21 have been shown to induce glucose uptake into adipocytes, and FGF21 treatment also resulted in acute suppression of adipocyte lipolysis and reduction in plasma free fatty acid levels [14,22,23].  These effects may directly contribute to the improvement in glucose regulation and insulin sensitivity.  Whether other mechanisms associated with adipose tissue also contribute remains to be explored.  The role of liver in glucose regulation remains an open question.  It has been shown that β-oxidation in liver was increased in FGF19 transgenic mice, which may have led to improvement in metabolic conditions and glucose homeostasis [12].  Recent evidence has also suggested that FGF21 may directly act on liver to regulate hepatic gluconeogenesis [24, 25].  Although our results suggest that liver FGFR4 activation may not be important in these activities, the roles for other liver-expressed FGFRs need to be further explored.  Alternatively, if liver is not a direct target tissue for FGF19 or FGF21, the identification of secondary signals emanating from other tissues to liver will be important in elucidating the overall mechanism that leads to the beneficial changes observed for this subfamily of FGF molecules.

In summary, FGF19 subfamily members are a unique group of molecules that are being actively studied.  Emerging data in recent years have allowed us to begin making connections between physiological phenotypes and the molecular details, such as receptor specificity and co-factor requirements.  In the future, new findings should allow us to fill in knowledge gaps and to gain better insight into the mechanisms of action of FGF19 subfamily members.
